# A laboratory task to assess epistemic mistrust: behavioral evidence for mediation between childhood trauma and borderline personality features in young adults

**DOI:** 10.1186/s40479-025-00333-z

**Published:** 2025-12-26

**Authors:** Elizabeth Li, Chloe Campbell, Linda C. Mayes, Georgia McRedmond, Patrick Luyten

**Affiliations:** 1 Research Department of Clinical, Educational and Health Psychology, University College London, London, England, UK; 2 Anna Freud Centre, London, England, UK; 3 Yale Child Study Center, Yale School of Medicine, New Haven, USA; 4 Faculty of Psychology and Educational Sciences, University of Leuven, Leuven, Belgium

**Keywords:** Epistemic trust, Behavioural assessment, BART-ET, ETMCQ, Epistemic disruptions, Social-cognitive vulnerability

## Abstract

**Background:**

Disruptions in epistemic trust have been recognised as key sequelae of trauma and as markers of vulnerability to borderline personality pathology. However, prior research has relied primarily on self-reports and lacks behavioural measures of epistemic stance. The present pre-registered studies introduce a novel behavioural task—the Balloon Analogue Risk Task for Epistemic Trust (BART-ET)—and examine its associations with borderline personality features, trauma history, and psychological distress.

**Methods:**

Two cross-sectional studies were conducted with a combined sample of 273 young adults aged 18–25 (Study 1: *N* = 120; Study 2: *N* = 153). Participants completed self-report measures of borderline personality features (PAI-BOR) and epistemic trust, mistrust, and credulity (ETMCQ). Study 2 additionally included the Childhood Trauma Questionnaire (CTQ) and the Brief Symptom Inventory (BSI-GSI). All participants completed the BART-ET in a laboratory setting, which operationalised epistemic mistrust as the degree of deviation from a confederate experimenter’s advice during a risk-taking task. Analyses involved correlational tests and structural equation modelling (SEM) to evaluate hypothesised associations and mediation pathways.

**Results:**

As expected, across both studies, higher levels of borderline personality traits were associated with greater epistemic mistrust—both behaviourally (on the BART-ET) and via self-report (ETMCQ)—and with greater epistemic credulity, but not with epistemic trust, as measured with the ETMCQ. Behavioural and self-report measures of mistrust were significantly correlated, suggesting convergent validity of the BART-ET as an index of epistemic mistrust. In Study 2, childhood trauma exposure was associated with borderline features and with epistemic mistrust assessed behaviourally and via self-report. Preregistered mediation models controlling for general distress (BSI-GSI) suggested that the association between childhood trauma and epistemic mistrust was not unique to BPD features.

**Conclusions:**

These findings suggest that epistemic mistrust—rather than a simple absence of trust—is a social-cognitive correlate of borderline personality vulnerability and trauma exposure in young adults. The results also indicate that the BART-ET may be a useful behavioural tool for studying epistemic mistrust in clinical contexts, though further validation is needed.

## Background

Borderline personality disorder (BPD) is a complex and impairing condition marked by chronic interpersonal instability, affective dysregulation, and identity disturbance [1]. Its prevalence is estimated at approximately 2% in the general population, with considerably higher rates in clinical settings (ranging from 10% to 50% among psychiatric patients and individuals seeking psychological support) [1]. One emerging theoretical account highlights the role of epistemic mistrust—an enduring tendency to distrust information conveyed by others—in the development and maintenance of BPD [2]. Epistemic trust, by contrast, is thought to develop within secure attachment relationships, where contingent, attuned caregiving fosters openness to social communication and learning. Epistemic trust allows individuals to internalise socially relevant knowledge and adapt to their environment through social learning [3]. In contrast, early interpersonal adversity—such as parental maltreatment—can disrupt attachment security and mentalizing abilities [4, 5], impairing the capacity to evaluate the trustworthiness of others’ communications. In such contexts, maladaptive epistemic strategies may emerge, including rigid scepticism toward others’ communications (i.e. epistemic mistrust) and overly passive acceptance of information (i.e. epistemic credulity). These strategies may initially serve as adaptive responses to inconsistent, abusive, or invalidating caregiving [6].

When generalized beyond the original context, however, entrenched mistrust or credulity can impede adaptive social learning. Heightened mistrust may lead individuals to reject even trustworthy or authoritative sources, instead favoring unverified alternatives that confirm pre-existing suspicions. Conversely, extreme credulity may manifest as an indiscriminate or overly trusting acceptance of interpersonal information, leaving one vulnerable to manipulation or misinformation due to insufficient scrutiny of the communicator’s intent. Over time, such disruptions in epistemic stance may undermine personality and social functioning—affecting identity integration, emotion regulation, and interpersonal boundaries—which are core impairments observed in borderline presentations [3, 7]. Indeed, BPD is often viewed as an exemplar of the clinical consequences of entrenched epistemic mistrust (and sometimes credulity), marked by volatile relationships and distorted social-cognitive processing [2].

Experimental evidence supports the association between BPD traits and social-cognitive disturbance. Individuals with BPD often show reduced trust in social interactions [8] and lower expectations of inclusion [9] and respond less cooperatively even when primed with positive social signals [10–12]. They are also more likely to interpret neutral or ambiguous social cues as threatening or untrustworthy [13–18]. A recent meta-analysis provides further support for these assumptions, reporting consistent evidence of heightened mistrust in individuals with BPD when assessed through laboratory-based tasks [19]. Interestingly, intranasal oxytocin— a neuropeptide usually known to enhance prosocial behaviour—failed to increase trust in individuals with BPD [20]. These patterns suggest a deep-seated disruption in their social information processing and capacity to engage in trusting interpersonal exchanges.

To date, empirical studies have relied on self-report to examine the role of epistemic stance in the relationship between childhood trauma and borderline personality features. Knapen et al. [21] found that epistemic trust mediated the link between childhood trauma and BPD features, although trust explained less variance than attachment or mentalizing. In a second study, Knapen et al. [22] showed that individuals with personality disorders, especially BPD, report lower epistemic trust than both healthy and clinical controls. Multidimensional models have been proposed by Kumpasoğlu et al. [23] and Schwarzer et al. [24], who modelled all three epistemic stance dimensions as mediators of the trauma–BPD link. Kumpasoğlu et al. [23] found that trauma predicted increased mistrust and credulity, but only these dimensions—not epistemic trust—significantly mediated the trauma–BPD link. Similarly, Schwarzer et al. [24] demonstrated that mistrust, but not trust, mediated the effects of trauma on personality functioning. These findings suggest that mistrust and credulity are more strongly linked to personality vulnerability than the mere absence of trust.

While disruptions in epistemic trust have been closely linked to borderline personality features, it remains unclear whether this association reflects a distinct interpersonal dysfunction characteristic of BPD or a broader, transdiagnostic marker of psychological distress. General distress has been associated with altered social-cognitive functioning [25], raising the possibility that elevated epistemic mistrust and credulity may emerge as non-specific responses to emotional dysregulation or general psychopathology [26]. Disentangling these possibilities is essential for clarifying the construct validity of epistemic stance and requires controlling for overall symptom severity. Additionally, dose–response evidence suggests that greater trauma exposure predicts more severe interpersonal dysfunction [27], highlighting the importance of examining whether epistemic mistrust and borderline pathology increase proportionally with trauma severity.

Yet, despite promising findings, little is still known about whether epistemic stance can be measured through behaviour—rather than self-perception—and whether such behavioural indicators align with self-reported epistemic stance and borderline personality traits. Existing research has relied exclusively on self-report measures, limiting the ecological validity of their conclusions and leaving open questions about how epistemic stance is enacted in real-time interpersonal contexts—how individuals respond to advice or social input under uncertainty. It also remains unclear whether epistemic mistrust reflects a social-cognitive construct specific to borderline personality vulnerability, or a broader transdiagnostic marker of psychological distress. Furthermore, no studies have tested whether epistemic stance patterns vary according to trauma severity, or whether epistemic mistrust uniquely mediates the impact of childhood trauma on borderline personality features when general psychological distress is controlled. Understanding these mechanisms is particularly important in young adults, a developmental period during which social learning continues to shape identity formation and interpersonal functioning [28, 29]. The present research was designed to address these gaps.

### The present study

The present research introduces the Balloon Analogue Risk Task – Epistemic Trust (BART-ET), a novel behavioural paradigm designed to assess epistemic stance by measuring advice-taking behaviour under uncertainty. Adapted from the original BART [30], which has been validated as a behavioural measure of risk-taking and risk-avoidant strategies [31, 32], the BART-ET repurposes the task to quantify the discrepancy between advice received and behaviour enacted, thereby indexing epistemic mistrust.

Two cross-sectional studies were conducted. Study 1 (*N* = 120) piloted the BART-ET and examined its associations with self-reported borderline personality traits and epistemic stance, using the Personality Assessment Inventory – Borderline Features Scale (PAI-BOR) and the Epistemic Trust, Mistrust, and Credulity Questionnaire (ETMCQ). Study 2 (*N* = 153) sought to address some limitations of Study 1 by including the Childhood Trauma Questionnaire (CTQ) and the Brief Symptom Inventory (BSI-GSI), enabling the examination of childhood trauma exposure, general psychological distress, and epistemic stance in relation to BPD features. Including the BSI-GSI also allowed us to test whether associations between epistemic stance and BPD reflect a distinct interpersonal dysfunction specific to BPD or a broader, transdiagnostic marker of distress.

Based on prior research, we pre-registered the following hypotheses. First, higher levels of borderline personality features (PAI-BOR) would be associated with greater advice resistance on the BART-ET, indexed by larger discrepancies between the experimenter’s suggestions and participants’ responses—as an expression of greater epistemic mistrust (Hypothesis 1). Second, borderline personality features would correlate positively with self-reported epistemic mistrust and credulity, and negatively with epistemic trust (ETMCQ), reflecting difficulties in appropriately evaluating others’ communications (Hypothesis 2). Third, behaviourally assessed epistemic mistrust (BART-ET scores) were expected to correspond with self-report measures, such that BART-ET scores would correlate positively with epistemic mistrust and negatively with epistemic trust (Hypothesis 3). Fourth, greater exposure to childhood trauma (CTQ) would be associated with higher BPD features and greater epistemic mistrust—both behavioural (BART-ET) and self-reported (ETMCQ)—as well as higher credulity and lower epistemic trust as captured by the ETMCQ subscales (Hypothesis 4, Study 2). These associations were expected to follow a dose–response pattern, such that increasing severity of childhood trauma would correspond to progressively more maladaptive epistemic stance scores and higher BPD-related traits. Finally, we hypothesised that epistemic mistrust would mediate the relationship between childhood trauma and borderline personality features, even when controlling for general distress (Hypothesis 5, Study 2). Mistrust was operationalised via both BART-ET scores and ETMCQ subscales, allowing comparison across behavioural and self-report indices.

## Methods

### Participants

Participants in both studies were recruited via the UCL Psychology Subject Pool and wider university platforms. In Study 1 (*N* = 120, October 2022–March 2023) and Study 2 (*N* = 153, March–April 2025), participants were young adults aged 18–25 (total *N* = 273). Across both samples, participants were predominantly Asian and female, and a substantial proportion reported recent emotional problems (37% in Study 1; 45% in Study 2). A full demographic breakdown is presented in Table 1.

**Table 1:** Participant demographics and mental health characteristics in study 1 and study 2

Characteristic	Study 1 (*N* = 120)	Study 2 (*N* = 153)
**Age**	20.06 (1.96)^1^	19.99 (1.85)^1^
**Sex**		
Female	93 (77%)	121 (79%)
Male	27 (23%)	32 (21%)
**Ethnicity** ^2^		
Asian	79 (65.8%)	80 (52%)
Black	7 (5.8%)	8 (5.2%)
White	28 (23.3%)	41 (27%)
Mixed	3 (2.5%)	12 (7.8%)
Other	3 (2.5%)	12 (7.8%)
**Emotional problems in the past 4 weeks**		
Yes	44 (37%)	69 (45%)
No	76 (63%)	75 (49%)
Prefer not to say	—	9 (5.9%)
**Rating of Mental Health**		
Excellent	15 (13%)	10 (6.5%)
Somewhat Good	51 (43%)	51 (33%)
Average	42 (35%)	66 (43%)
Somewhat Poor	9 (7.5%)	26 (17%)
Poor	3 (2.5%)	—
**Psychological treatment (current or past)**		
Yes	30 (25%)	43 (28%)
No	90 (75%)	103 (67%)
Prefer not to say	—	7 (4.6%)
**Current medication use for mental health conditions**		
Yes	5 (4.2%)	7 (4.6%)
No	107 (89%)	145 (95%)
Prefer not to say	8 (6.7%)	1 (0.7%)
**Socio-economic background** ^3^		
Professional background	—	103 (67%)
Intermediate background	—	18 (12%)
Lower socio-economic background	—	21 (14%)
Prefer not to say	—	11 (7.2%)

^1^Mean (SD)

^2^Ethnicity categories are grouped for reporting purposes

^3^Collected in Study 2 only. Socio-economic background assessed via occupation of main household earner at age 14

The combined sample size exceeded the minimum identified through a priori power analyses and Monte Carlo simulations [33, 34], providing ≥ 80% power to detect moderate effects (*r* = .30, d = 0.50) across correlational, group comparison, and mediation analyses [35]. Both studies received ethical approval from the UCL Research Ethics Committee (18057.001 for Study 1; 0466 for Study 2), and all participants provided informed consent. Participants received course credit or monetary compensation; incentive amount was included as a covariate in sensitivity analyses, which indicated that findings were robust to differences in compensation.

### Procedures

All participants first read an online information sheet and provided written informed consent before completing a battery of self-report questionnaires hosted on Qualtrics. In Study 1, participants completed demographic questions, the Personality Assessment Inventory – Borderline Features Scale (PAI-BOR), and the Epistemic Trust, Mistrust, and Credulity Questionnaire (ETMCQ), which took approximately 5 min. In Study 2, an expanded battery was administered, including the same measures as Study 1 along with the Childhood Trauma Questionnaire (CTQ) and the Brief Symptom Inventory (BSI), and took approximately 15 min. To ensure data quality in the self-report measures, two attention check items were embedded in the questionnaire battery. These instructed participants to select a specific response (e.g., “Please select ‘3 (Very true)’ to show that you are reading the questions carefully”). All participants passed the attention checks, and no invariant response patterns were detected.

Following the online questionnaires, participants were scheduled for an in-person session via direct email. Upon arrival at the UCL Psychology Laboratories, they were welcomed by a trained experimenter and escorted to an individual cubicle for the laboratory task. The laboratory task was the Balloon Analogue Risk Task – Epistemic Trust version (BART-ET), which lasted approximately 25 min (for a full description, see below). For the in-person task, the experimenter remained present throughout to monitor comprehension and engagement. Given the interactive format—which required responses on every trial—and the task’s relatively brief duration, the risk of inattentiveness was considered minimal. Overall participation lasted approximately 30 min in Study 1 and 40 min in Study 2.

### Measures

#### Demographic and mental health characteristics

Participants reported age, sex, ethnicity, along with mental health-related information in both studies. These included whether they had experienced emotional problems affecting daily life in the past four weeks, their overall self-rated mental health, whether they had received or were currently receiving psychological treatment, and any current use of psychiatric medication. In Study 2, an additional item assessed socio-economic background based on parental occupation, with response options including professional, intermediate, and lower socio-economic backgrounds.

#### Personality Sssessment Inventory – Borderline Features Scale (PAI-BOR)

The PAI-BOR is a 24-item self-report measure of borderline personality features in nonclinical populations [36]. It comprises four subscales—affective instability, identity problems, negative relationships, and self-harm—each with six items. Responses are rated on a 4-point scale (0 = false to 3 = very true). A total score of 38 or higher suggests clinically significant borderline features. The scale has demonstrated excellent internal consistency (*α* > 0.80) and construct validity in diverse populations [37]. In the current study, internal consistency was *α* = 0.90 in Study 1 and *α* = 0.86 in Study 2.

#### Epistemic Trust, Mistrust, and Credulity Questionnaire (ETMCQ)

The ETMCQ is a 15-item self-report measure assessing individuals’ tendencies to accept, reject, or over-accept interpersonal information [6]. It comprises three 5-item subscales: epistemic trust (e.g., “I usually ask people for advice when I have a personal problem”), epistemic mistrust (e.g., “I often feel that people do not understand what I want and need”), and epistemic credulity (e.g., “I am often considered naïve because I believe almost anything that people tell me”). Items are rated on a 7-point scale (1 = strongly disagree to 7 = strongly agree). The scale has demonstrated acceptable reliability and validity in community samples [6]. In the current study, internal consistency in Study 1 was *α* = 0.71 (Trust), *α* = 0.66 (Mistrust), and *α* = 0.78 (Credulity); in Study 2, *α* = 0.75 (Trust), *α* = 0.55 (Mistrust), and *α* = 0.79 (Credulity).

#### Childhood Trauma Questionnaire (CTQ)

The CTQ is a 28-item retrospective self-report measure of childhood maltreatment across five domains: emotional abuse, physical abuse, sexual abuse, emotional neglect, and physical neglect [38]. Items are rated on a 5-point Likert scale (1 = never true to 5 = very often true). Clinically validated cut-offs are available to classify levels of exposure [38]. For emotional abuse, scores of 13 or more indicate moderate exposure, and scores of 18 or more indicate severe exposure. For physical abuse, scores of 10 or more suggest moderate exposure, and 13 or more indicate severe exposure. For sexual abuse, moderate exposure is defined as a score of 8 or above, and severe exposure as 13 or above. For emotional neglect, scores of 15 or more represent moderate exposure, while scores of 18 or more indicate severe exposure. For physical neglect, scores of 10 or more suggest moderate exposure, and scores of 15 or more indicate severe exposure. The CTQ has demonstrated strong psychometric properties in both clinical and nonclinical samples, with internal consistency coefficients ranging from *α* = 0.63 to 0.95 across subscales and criterion-related validity correlations of *r* = .50–0.75 [39, 40]. In the current study (Study 2), internal consistency was *α* = 0.91.

#### Brief Symptom Inventory – Global Severity Index (BSI-GSI)

The BSI is a 53-item self-report measure of psychological distress across nine symptom dimensions and three global indices [41]. In the current study, only the Global Severity Index (GSI) was used as an overall index of current distress. Participants rated how much each symptom distressed them over the past seven days on a 5-point scale (0 = not at all to 4 = extremely). The GSI has demonstrated excellent internal consistency (*α* > 0.80) and robust construct and convergent validity across clinical and nonclinical samples [41]. According to standard interpretive guidelines, T scores below 55 are considered within the normal range; T scores of 55–59 indicate mild psychological distress; T scores of 60–64 reflect moderate or borderline clinical distress; and T scores of 65 or above suggest clinically significant levels of distress [41]. In the current study (Study 2), the BSI-GSI demonstrated excellent internal consistency (*α* = 0.96).

#### Balloon Analogue Risk Task – Epistemic Trust (BART-ET)

The Balloon Analogue Risk Task – Epistemic Trust (BART-ET) is a computerised, laboratory-based task designed to assess epistemic mistrust in terms of the deviation from trustworthy advice. The task yields a single behavioural score, with higher scores indicating greater epistemic mistrust.

The BART-ET is adapted from the original Balloon Analogue Risk Task (BART) [30], which was designed to measure risk-taking. In the original BART, each trial presents a balloon that participants inflate by clicking a “PUMP” button. With each pump, the balloon expands and accumulates points, but may explode at an unpredictable threshold. Participants may choose to stop pumping and bank the points at any time. If the balloon explodes, all points for that trial are lost, though previously banked points remain safe. The task therefore requires participants to balance risk and reward to maximise their overall score.

The BART-ET repurposes this format to assess advice-taking behaviour under uncertainty, rather than risk-taking. For a visual overview of the whole procedure, including the BART-ET structure, see Fig. 1a. Participants complete two rounds of 30 trials, spaced approximately 10 min apart. In the first round (see Fig. 1b), 21 of 30 balloons (70%) are programmed to explode regardless of the number of pumps entered, producing repeated negative outcomes. Following this, participants see the message on the screen: *“Be careful! So far*,* you had 21 balloons explode*,* and your earned points are lower than 73% of players in this game. Let’s try another round of 30 balloons.”* Immediately afterward, the experimenter delivers a scripted verbal prompt: *“I’m sorry that you didn’t do very well in the first round. However*,* we have another round of 30 balloons. I have played this game several times myself before—how about we play together in the second round? I’ll give you my advised pump number for each balloon*,* but there’s no pressure to follow it. Let’s play together?”* This scripted intervention is designed to simulate a realistic interpersonal interaction, introducing advice from a seemingly experienced and knowledgeable other while preserving the participant’s autonomy. It serves to test openness to interpersonally transmitted knowledge in a context of uncertainty and prior failure (i.e., epistemic trust).

**Fig. 1a:**
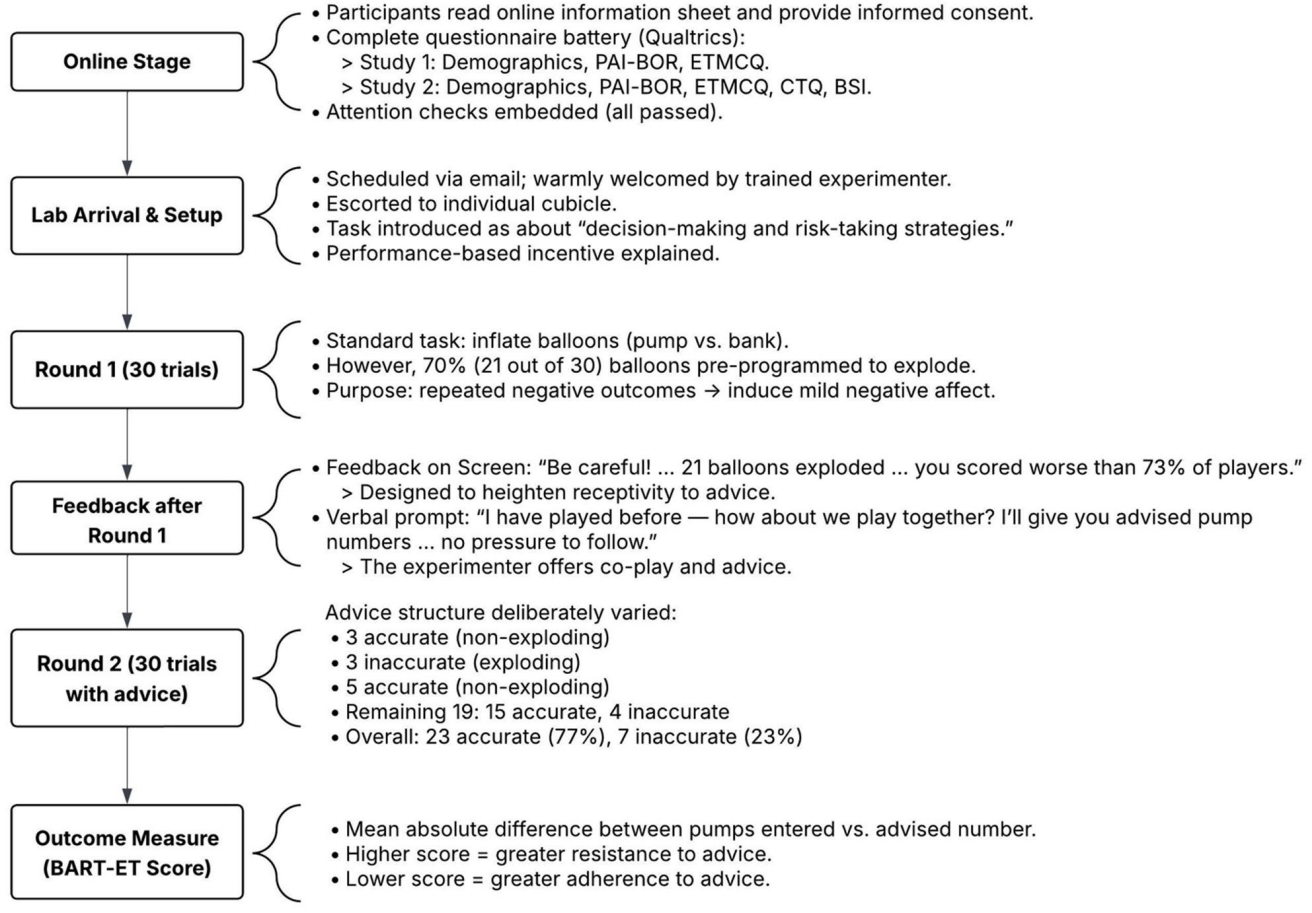
Timeline of the data collection

**Fig. 1b:**
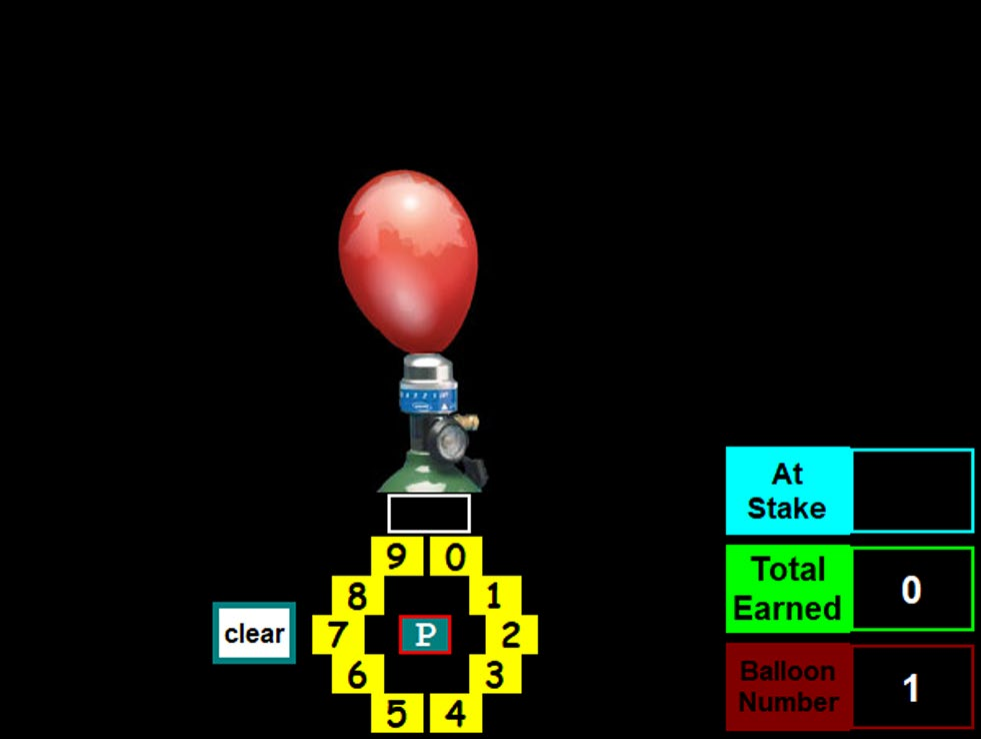
BART-ET interface during Round 1 (no advice). Note. The interface displays the virtual balloon and pump controls. Participants complete the first round independently, with 70% of balloons programmed to explode, leading to repeated negative outcomes. This setup establishes a context of uncertainty and failure prior to the introduction of external input in Round 2

**Fig. 1c:**
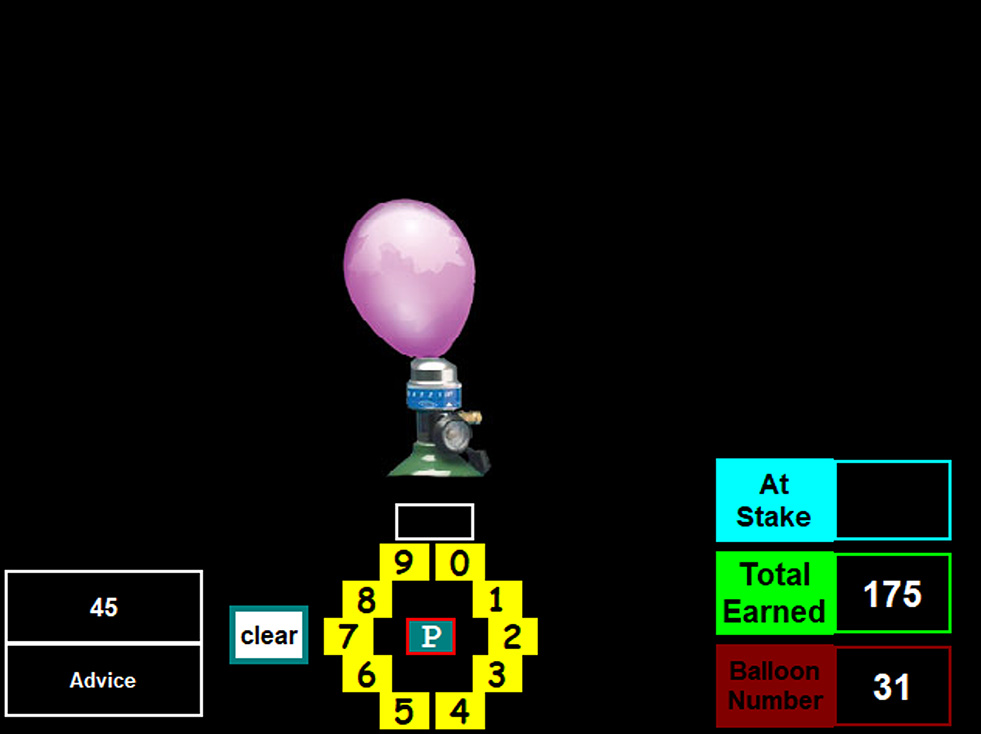
BART-ET interface during Round 2 (with advised pump numbers). Note. The interface displays the experimenter’s suggested number of pumps alongside the virtual balloon and pump controls. Advised numbers appear on screen three seconds after each trial begins, simulating interpersonal guidance while preserving participant autonomy. To support this impression, the experimenter visibly enters the advised number on their own device, creating the appearance of playing the task alongside the participant. This setup models advice-taking behaviour, allowing participants to follow, ignore, or deviate from the given recommendation

During the second round (see Fig. 1c), advice accuracy is deliberately varied to create an “early violation” of epistemic trustworthiness, modelled after the Trust task used in King-Casas et al. [8]. Pilot versions of the task without this manipulation did not appear to activate epistemic trust processes as effectively, whereas the current version was more successful in eliciting responses consistent with epistemic trust activation. Specifically, the experimenter provides correct advice for the first three non-exploding trials, then gives inaccurate advice for three exploding trials, followed by five accurate non-exploding trials. The remaining 19 trials include 15 accurate and 4 inaccurate pieces of advice. In total, advice is correct for 23 out of 30 trials (76.7%) and incorrect for seven (23.3%). The BART-ET score is calculated as the mean absolute difference between the participant’s entered pump number and the experimenter’s advised number across all 30 trials in the second round, regardless of whether the participant pumped more or less. Higher BART-ET scores thus reflect greater resistance to advice as an index of epistemic mistrust, while lower BART-ET scores reflect lower resistance and closer adherence to the guidance received, suggesting lower epistemic mistrust.

Moreover, participants were told the task measured decision-making and risk-taking strategies. To disguise the true aim of the task, an additional task modification was included: balloon colour varied across trials. This encouraged participants to infer a (non-existent) relationship between balloon colour and explosion probability, leading them to focus on superficial features rather than the role of advice. This misdirection was intended to reduce demand characteristics and enhance the ecological validity of the advice-taking context. To encourage engagement, participants were informed that compensation included a performance-based incentive: in Study 1, a base payment of £3 and a bonus of up to £2 based on task performance (totalling £5); in Study 2, a base payment of £5 and a potential bonus of £3 (totalling £8). In practice, all participants received the full amount, regardless of performance. After completing the BART-ET, participants were asked to guess the true purpose of the task. Most believed the study concerned decision-making, risk-taking, or strategy optimisation; others mentioned social influence, bias, or conformity. No participants correctly identified epistemic trust as the study’s focus. Finally, participants were fully debriefed and provided with a written debriefing sheet explaining the use of deception and listing support services. No participants reported emotional distress.

### Statistical analysis

Analyses were conducted in RStudio (v4.2.1). Descriptive statistics summarised sample characteristics. Normality was assessed using Shapiro–Wilk tests; where assumptions were violated, non-parametric alternatives (Spearman’s ρ) were used. Bonferroni corrections were applied within each hypothesis set, with adjusted α levels reported. Missing data were minimal, and all analyses were run on complete cases. To account for potential motivational effects, compensation amount was included as a covariate in sensitivity checks conducted in response to reviewer comments.

Pre-registered analyses first examined associations between borderline personality features (PAI-BOR total score) and BART-ET discrepancy scores (Hypothesis 1). The discrepancy score indexed epistemic mistrust operationalised as the mean absolute deviation from advice, with larger values approximating greater resistance. We then tested correlations between PAI-BOR and self-reported epistemic stance on the ETMCQ (Hypothesis 2), expecting positive associations with mistrust and credulity, and negative associations with trust. To evaluate convergent validity, BART-ET scores were correlated with ETMCQ subscales (Hypothesis 3), with the prediction that behavioural indices of mistrust would align with self-reports.

In Study 2, we extended analyses to childhood trauma (CTQ total score), testing its associations with epistemic stance (BART-ET and ETMCQ), borderline traits (PAI-BOR), and general distress (BSI-GSI) (Hypothesis 4). Dose–response associations were further explored descriptively using CTQ severity categories and visualised with bar charts. Finally, preregistered structural equation models (SEMs) tested whether epistemic mistrust mediated the relationship between childhood trauma and borderline traits, controlling for general distress (BSI-GSI) (Hypothesis 5). Mistrust was modelled using two separate manifest indicators: (1) behavioural mistrust, assessed via BART-ET discrepancy scores, and (2) self-reported mistrust, assessed via the ETMCQ Mistrust subscale. Each indicator was tested in an independent single-mediator model examining indirect pathways from childhood trauma to borderline personality features. All models were just-identified (df = 0); therefore, no global fit indices were reported, and inference was restricted to path-level estimates with bootstrapped (5,000 resamples) confidence intervals. Non-normality was addressed using the robust MLR estimator. Exploratory, non-registered extensions included parallel-outcome SEMs treating BSI-GSI as an additional outcome rather than a covariate.

## Results

### Descriptive statistics

Descriptive statistics for variables in Study 1 are presented in Table 2a and for Study 2 in Table 2b. For the BART-ET, mean discrepancy scores between the advice given and the actual number of pumps were comparable across studies. PAI-BOR scores were slightly higher in Study 2 (M = 27.63, SD = 10.48) than in Study 1 (M = 25.67, SD = 11.99). ETMCQ subscale scores were similar across the two samples.

**Table 2a:** Descriptive statistics and spearman correlations for variables in study 1

	1	2	3	4	5
1 BART-ET	—				
2 PAI-BOR	0.27**	—			
3 ETMCQ Trust	-0.11	-0.06	—		
4 ETMCQ Mistrust	0.33***	0.43***	-0.20*	—	
5 ETMCQ Credulity	0.18	0.43***	0.29**	0.17	—
Mean (SD)	14.26 (8.02)	25.67 (11.99)	26.67 (4.54)	20.96 (4.89)	16.58 (5.90)
Median (IQR)	13.00 (10.07)	23.5 (16.25)	27.00 (7.25)	21.00 (6.00)	16.5 (8.25)
Range	1–36.9	5–67	13–35	9–33	5–34
Cronbach’s Alpha	—	0.899	0.707	0.659	0.778

Note. BART-ET = Balloon Analogue Risk Task for Epistemic Trust; PAI-BOR = Personality Assessment Inventory – Borderline Features Scale; ETMCQ = Epistemic Trust, Mistrust, and Credulity Questionnaire. p < .05 = *; *p* < .01 = **; *p* < .001 = ***

**Table 2b:** Descriptive statistics and spearman correlations for variables in study 2

	1	2	3	4	5	6	7
1 BART-ET	—						
2 PAI-BOR	0.44***	—					
3 ETMCQ Trust	0.06	0.09	—				
4 ETMCQ Mistrust	0.40***	0.40***	-0.33***	—			
5 ETMCQ Credulity	0.07	0.37***	0.18*	0.1	—		
6 CTQ Total	0.19*	0.46***	-0.13	0.28***	0.23**	—	
7 BSI-GSI	0.29***	0.67***	0.06	0.28***	0.27***	0.44***	—
Mean (SD)	14.86 (9.49)	27.63 (10.48)	26.53 (4.24)	21.5 (4.13)	16.67 (6.25)	42.45 (10.25)	60.50 (14.75)
Median (IQR)	13.53 (11.35)	28.00 (12.00)	27.00 (5.00)	22.00 (5.00)	16.00 (8.00)	41.00 (14.00)	56.67 (17.52)
Range	0–46.87	3–58	14–35	12–32	5–31	28–96	39.94–100.59
Cronbach’s Alpha	—	0.856	0.752	0.550	0.792	0.910	0.961

Note. BART-ET = Balloon Analogue Risk Task for Epistemic Trust; PAI-BOR = Personality Assessment Inventory – Borderline Features Scale; ETMCQ = Epistemic Trust, Mistrust, and Credulity Questionnaire; CTQ = Childhood Trauma Questionnaire; BSI-GSI = Brief Symptom Inventory – Global Severity Index. CTQ and BSI-GSI were administered in Study 2 only. p < .05 = *; *p* < .01 = **; *p* < .001 = ***

In Study 2, the CTQ total score averaged M = 42.45 (SD = 10.25), comparable to community samples reported in a large multinational study of over 19,000 participants across 24 international sites (M = 38.78, SD = 14.98) and lower than patient samples (M = 45.91, SD = 18.79) [42]. The BSI-GSI raw scores averaged M = 0.96 (SD = 0.58), with T-scores averaging M = 60.50 (SD = 14.75). Given that T-scores in the 60–64 range fall within the borderline clinical band, participants in this sample reported, on average, clinically relevant levels of distress.

### Assumption checks

Normality was tested using Shapiro–Wilk statistics. In Study 1, BART-ET, PAI-BOR, and epistemic trust were non-normally distributed (all *p* < .05). In Study 2, BART-ET, epistemic trust, credulity, CTQ, and BSI-GSI all deviated significantly from normality (all *p* < .001). Although log- and square-root transformations were attempted, several distributions (e.g., CTQ, BART-ET) remained skewed. For consistency, all correlations are reported using Spearman’s ρ. In all cases, results were consistent across non-parametric and parametric tests.

### Behavioural epistemic mistrust and BPD features (Hypothesis 1)

Consistent with preregistered predictions, higher levels of borderline personality features (PAI-BOR) were associated with greater behavioural epistemic mistrust as measured with the BART-ET in both studies (ρ = 0.27, *p* = .003 and ρ = 0.44, *p* < .001, in Study 1 and 2. respectively). These findings replicated across samples, supporting the hypothesis that borderline traits are linked to greater epistemic mistrust in uncertain contexts. The scatterplot in Fig. 2a illustrates the positive association between behavioural epistemic mistrust (BART-ET) and borderline personality features (PAI-BOR) across both studies.

**Fig. 2a:**
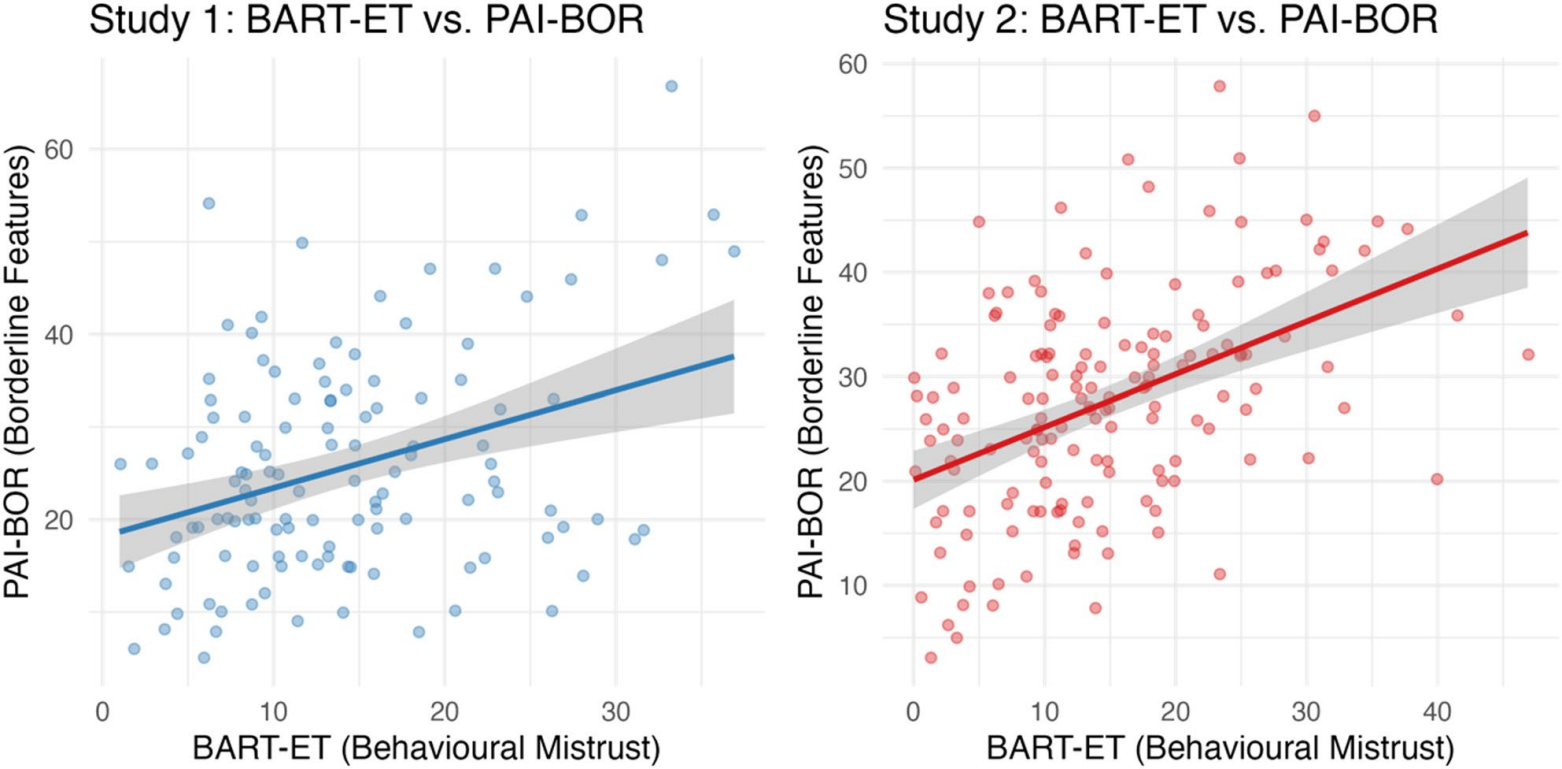
Association between behavioural epistemic mistrust (BART-ET) and borderline personality features (PAI-BOR)

**Fig. 2b:**
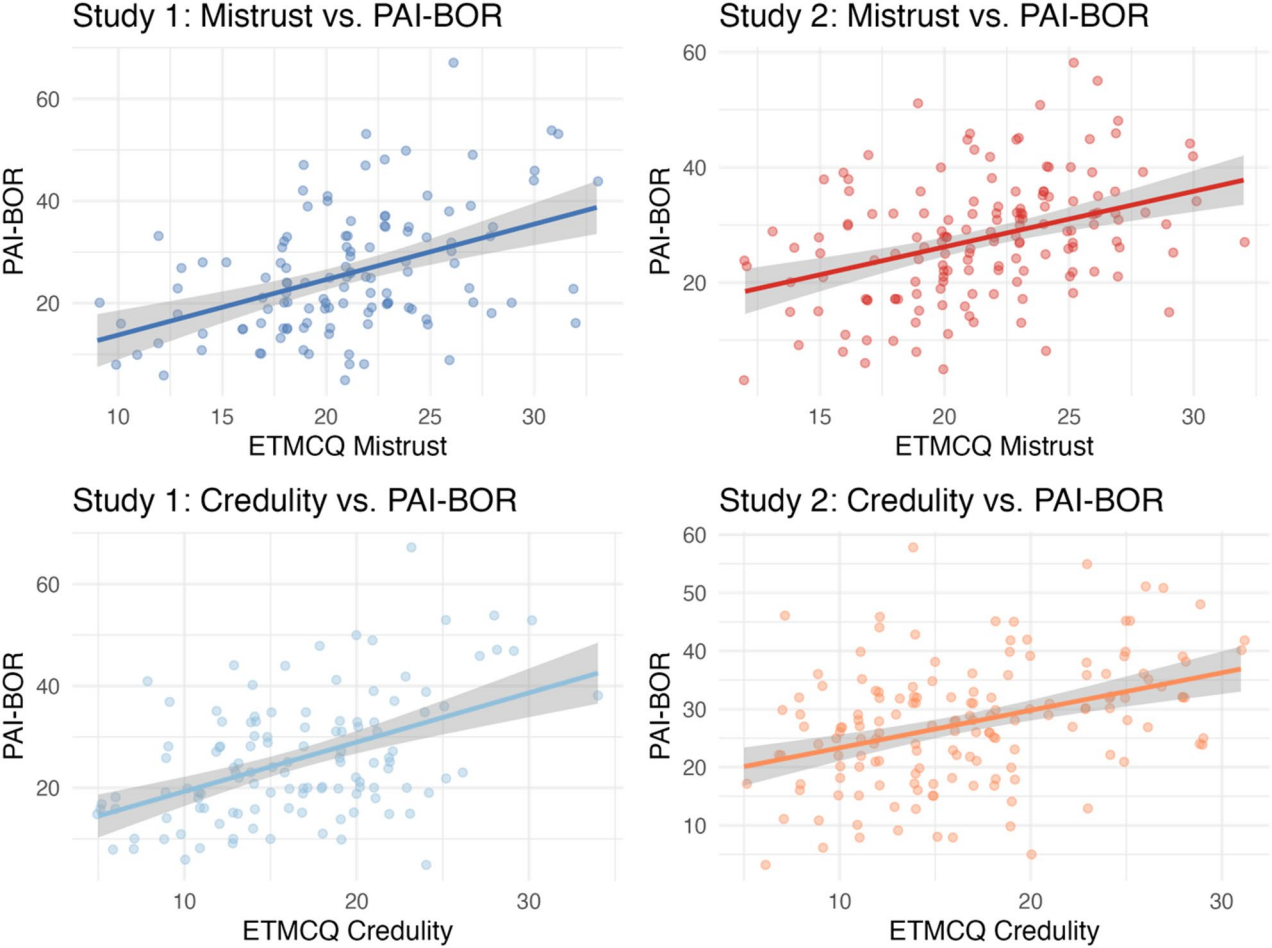
Associations between self-reported epistemic stance (ETMCQ mistrust and credulity) and borderline personality features (PAI-BOR)

**Fig. 2c:**
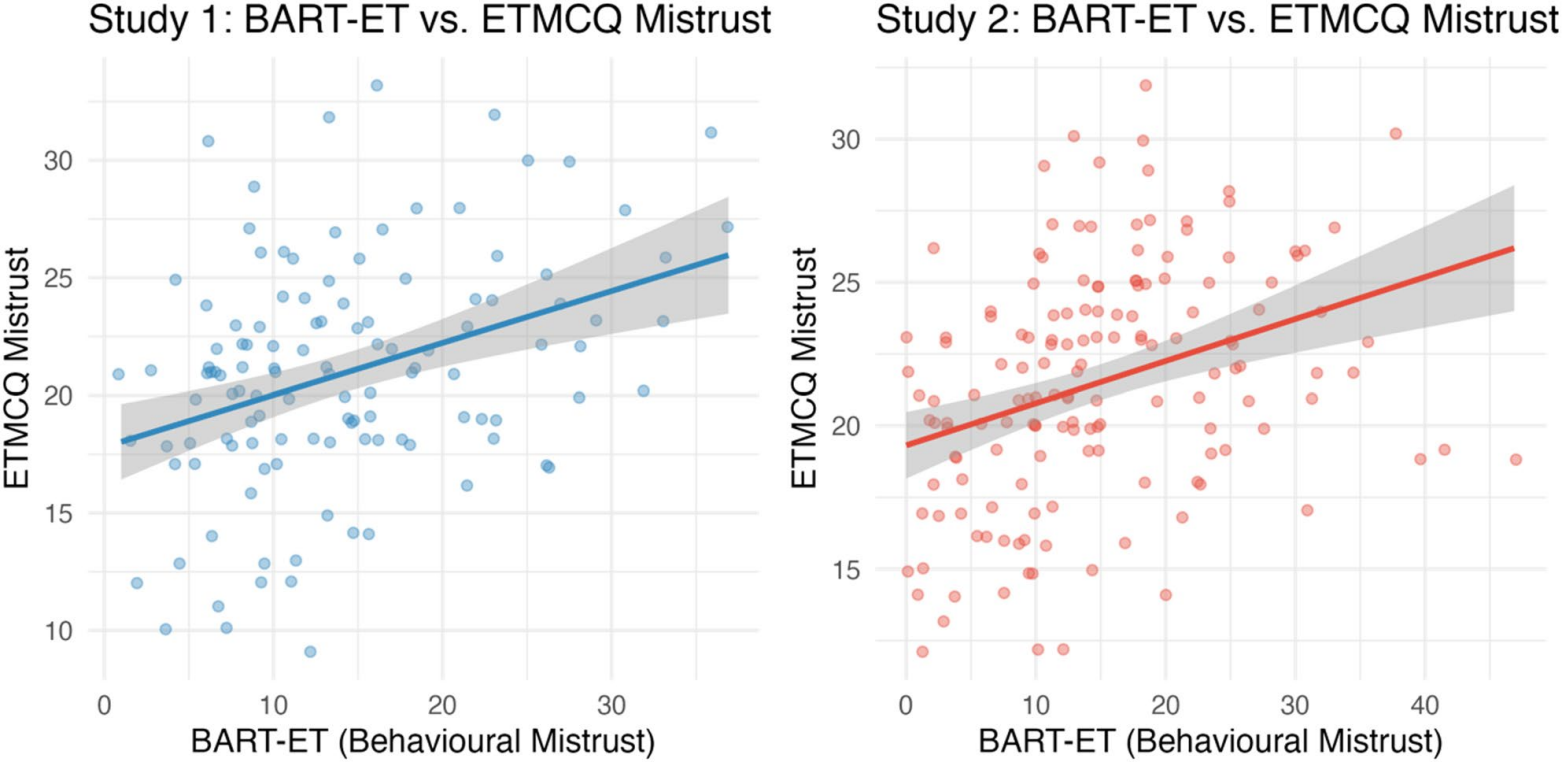
Concordance between behavioural epistemic mistrust (BART-ET) and self-reported mistrust (ETMCQ)

**Fig. 2d:**
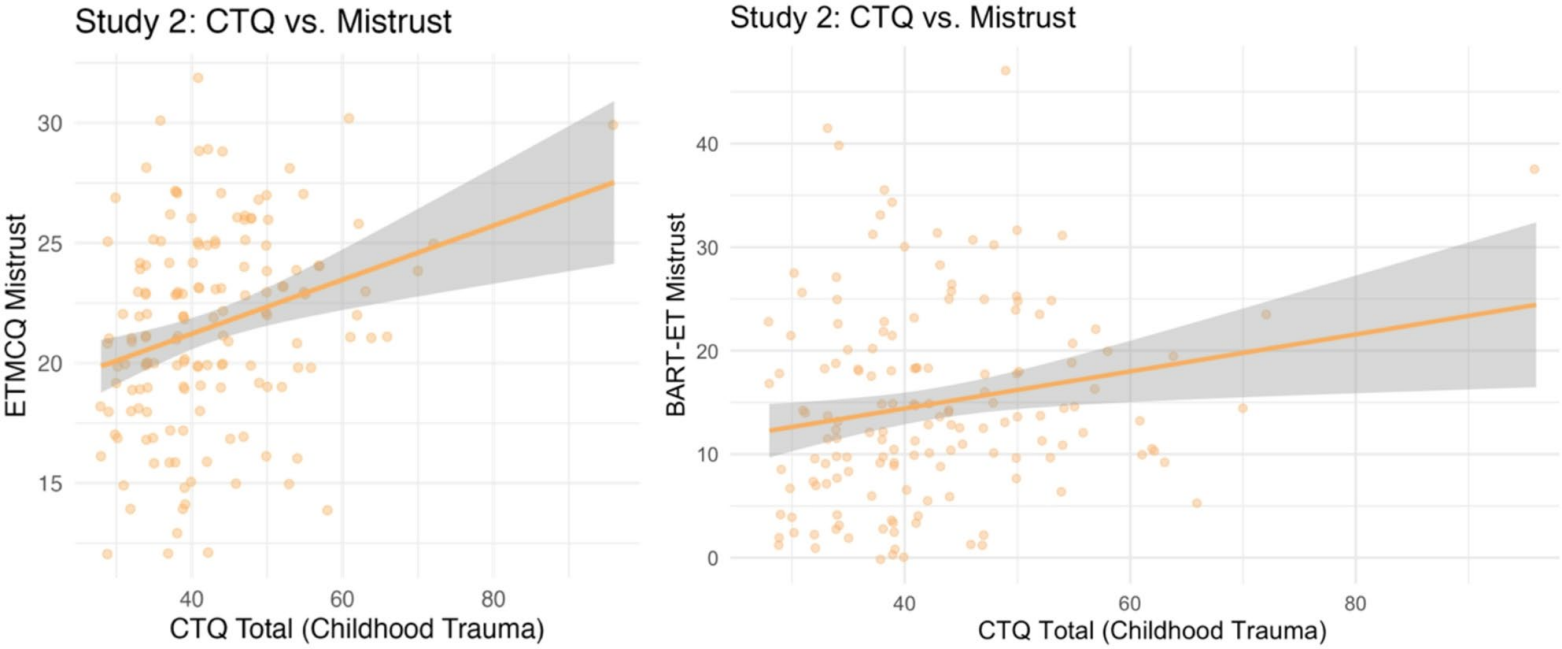
Associations between childhood trauma (CTQ) and epistemic mistrust (ETMCQ and BART-ET)

### Self-reported epistemic stance and BPD features (Hypothesis 2)

As predicted, PAI-BOR scores were positively correlated with self-reported mistrust and credulity in both studies. In Study 1, correlations with epistemic mistrust and credulity were each ρ = 0.43 (both *p* < .001). These associations were replicated in Study 2, with PAI-BOR correlating with both epistemic mistrust (ρ = 0.40, *p* < .001) and epistemic credulity (ρ = 0.37, *p* < .001). Contrary to expectations, PAI-BOR was not significantly associated with epistemic trust in either study. After Bonferroni correction (α ≈ 0.017), the significant positive associations with mistrust and credulity remained robust across both samples. Together, these findings provide further evidence that borderline features are associated with heightened mistrust and credulity, but not by reduced trust (see also Fig. 2b).

### Concordance between behavioural and self-report measures (Hypothesis 3)

BART-ET scores were significantly associated with self-reported mistrust across both studies (Study 1: ρ = 0.33, *p* < .001; Study 2: ρ = 0.40, *p* < .001). No other significant associations between behavioural and self-report measures emerged. After Bonferroni correction (α = 0.025), the correlation with mistrust remained significant in both samples. These findings indicate that the behavioural measure captures the mistrust dimension specifically, rather than being reducible to low levels of epistemic trust or epistemic credulity. Scatterplot shows the concordance between behavioural (BART-ET) and self-reported mistrust (ETMCQ), highlighting that both measures capture the mistrust dimension consistently (Fig. 2c).

### Childhood trauma, epistemic stance, and BPD features (Hypothesis 4, Study 2)

**Fig. 3:**
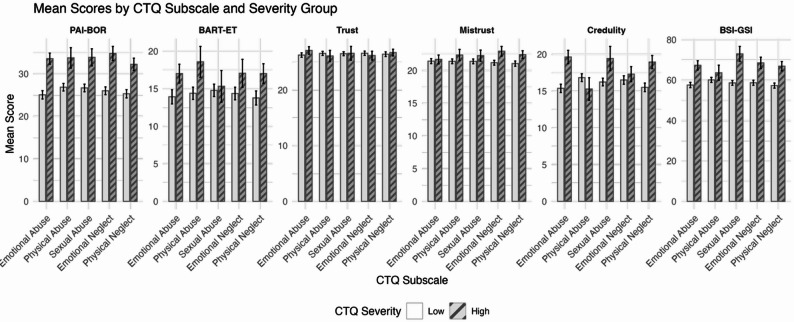
Bar Chart Showing Dose–Response Relationships of Psychological Outcomes by Childhood Trauma Exposure (Low vs. High) Across CTQ Subscales in Study 2. Note. CTQ = Childhood Trauma Questionnaire; PAI-BOR = Personality Assessment Inventory – Borderline Features Scale; BART-ET = Balloon Analogue Risk Task – Epistemic Trust; ETMCQ = Epistemic Trust, Mistrust, and Credulity Questionnaire; BSI-GSI = Brief Symptom Inventory – Global Severity Index. Trauma exposure levels were dichotomised using clinical cut-offs (Bernstein & Fink, 1998), with moderate and severe scores combined into a single “high exposure” group. Participants were grouped into low and high trauma exposure groups for each CTQ subscale. Bars represent mean scores for measure outcomes. Error bars represent standard deviations

Pre-registered hypotheses focused on trauma subtypes (e.g., emotional vs. physical vs. sexual abuse/neglect) in relation to epistemic stance and borderline features. Exploratory subscale analyses (Table 3; Fig. 3) compared outcomes across trauma exposure groups. Because cell sizes were too small in the “severe” tertile (*n* < 3), trauma subscales were dichotomised by combining moderate and severe into a single “high exposure” category. Wilcoxon rank-sum tests were then used to compare BART-ET, ETMCQ subscales, PAI-BOR, and BSI-GSI scores between trauma groups.

**Table 3:** Mean scores and Wilcoxon test results comparing low vs. High childhood trauma groups across outcomes

CTQ Subscale	Outcome Variable	Mean (Low)	Mean (High)	W	*p*	Sig
Emotional Abuse	PAI-BOR	25.1	33.6	1241.0	< 0.001	***
	BART-ET	13.9	17.0	1922.5	0.032	*
	ETMCQ Trust	26.3	27.1	2211.5	0.320	
	ETMCQ Mistrust	21.4	21.7	2312.0	0.553	
	ETMCQ Credulity	15.4	19.7	1512.5	< 0.001	***
	BSI-GSI	57.6	67.3	1535.0	< 0.001	***
Physical Abuse	PAI-BOR	26.9	33.8	712.5	0.010	*
	BART-ET	14.4	18.6	814.0	0.047	*
	ETMCQ Trust	26.6	26.1	1224.0	0.694	
	ETMCQ Mistrust	21.4	22.4	1003.5	0.376	
	ETMCQ Credulity	16.8	15.3	1353.0	0.253	
	BSI-GSI	60.1	63.6	996.0	0.354	
Sexual Abuse	PAI-BOR	26.7	33.9	777.5	0.003	**
	BART-ET	14.8	15.3	1296.0	0.856	
	ETMCQ Trust	26.5	26.6	1277.5	0.778	
	ETMCQ Mistrust	21.4	22.2	1172.5	0.394	
	ETMCQ Credulity	16.2	19.4	1005.5	0.079	†
	BSI-GSI	58.6	73.0	668.5	< 0.001	***
Emotional Neglect	PAI-BOR	26.0	34.8	877.5	< 0.001	***
	BART-ET	14.4	17.0	1472.5	0.191	
	ETMCQ Trust	26.6	26.2	1856.5	0.616	
	ETMCQ Mistrust	21.2	22.9	1285.5	0.028	*
	ETMCQ Credulity	16.5	17.3	1560.0	0.370	
	BSI-GSI	58.7	68.6	1044.5	< 0.001	***
Physical Neglect	PAI-BOR	25.3	32.3	1650.0	< 0.001	***
	BART-ET	13.8	17.0	2036.5	0.029	*
	ETMCQ Trust	26.4	26.7	2493.0	0.676	
	ETMCQ Mistrust	21.0	22.4	2038.5	0.029	*
	ETMCQ Credulity	15.5	19.0	1808.0	0.002	**
	BSI-GSI	57.3	66.9	1713.0	< 0.001	***

Note. Low indicates low trauma exposure. High includes both moderate and severe trauma exposure levels. W = Wilcoxon rank-sum statistic. Significance levels: p < .05 = *; p < .01 = **; p < .001 = ***; † indicates trend-level result (p < .10). A Bonferroni-corrected threshold of α = 0.002 was applied to adjust for multiple comparisons

BART-ET scores were significantly higher in the high trauma group for emotional abuse (*p* = .032), physical abuse (*p* = .047), and physical neglect (*p* = .029), suggesting greater behavioural epistemic mistrust among participants with these trauma histories. However, none of these effects survived Bonferroni correction (α ≈ 0.002). Self-reported mistrust was elevated in participants reporting emotional neglect (*p* = .028) and physical neglect (*p* = .029), but these associations also failed to survive Bonferroni correction. In contrast, credulity was significantly higher in participants exposed to emotional abuse (*p* < .001) and physical neglect (*p* = .002), and both effects remained significant after correction.

More robust and consistent effects were observed for PAI-BOR and BSI-GSI, which were significantly higher in the high trauma group when total CTQ scores were used as the grouping variable (ps < 0.001). Exploratory subscale comparisons also revealed elevated PAI-BOR and BSI-GSI scores across all CTQ subscales (all ps < 0.001), and all of these effects remained significant after Bonferroni correction. These findings suggest that childhood trauma is reliably linked with borderline features and general distress, whereas associations with behavioural and self-reported mistrust were smaller.

Non-registered exploratory analyses focused on associations between CTQ total scores and psychological outcomes in Study 2. Due to significant skew and kurtosis (skew = 1.47, kurtosis = 7.09), Spearman’s ρ was used. As shown in Table 2b, higher CTQ scores were significantly associated with greater ETMCQ mistrust (ρ = 0.28, *p* < .05), credulity (ρ = 0.23, *p* = .004), and borderline traits (ρ = 0.46, *p* < .001). After Bonferroni correction (α = 0.01), these associations remained significant. The association between CTQ scores and BART-ET discrepancy was smaller and did not reach significance (ρ = 0.19, *p* = .09). Scatterplots illustrate the associations between childhood trauma severity and both self-reported (ETMCQ) and behavioural (BART-ET) mistrust (Fig. 2d).

### Mediation of childhood trauma and BPD features via epistemic mistrust (Hypothesis 5, Study 2)

Spearman correlations indicated that BSI-GSI was positively associated with BART-ET (ρ = 0.29, *p* < .001), ETMCQ mistrust (ρ = 0.28, *p* < .001), and credulity (ρ = 0.27, *p* < .001). These findings suggested that mistrustful and credulous epistemic stances are broadly linked with general distress. Given these results, BSI-GSI was included as a covariate in subsequent mediation models.

Bootstrapped structural equation models were conducted as described in the Statistical Analysis section to test whether epistemic mistrust mediated the relationship between childhood trauma and borderline personality features. Epistemic mistrust was represented by behavioural (BART-ET) and self-reported (ETMCQ Mistrust) indicators in two separate models. Results of preregistered and exploratory mediation analyses are summarised in Tables 4a–4d and illustrated in Fig. 4a and d. Indirect effects, standard errors, and bootstrapped confidence intervals are reported for each model.

**Table 4a:** Standardised estimates and 95% confidence intervals (CI) of each path in the SEM testing BART–ET as a mediator between childhood trauma and borderline personality features, controlling for general psychological distress

Path	B	95% CI	*p*-Value	β
Path a: CTQ Total to BART-ET	0.07	-0.09 to 0.25	0.370	0.08
Path d1: BSI-GSI to BART-ET	4.01	1.72 to 6.46	< 0.001	0.26
Path b: BART-ET to PAI-BOR	0.31	0.17 to 0.46	< 0.001	0.28
Path c: CTQ Total to PAI-BOR	0.22	0.07 to 0.39	0.008	0.21
Path d2: BSI-GSI to PAI-BOR	7.88	5.61 to 10.08	< 0.001	0.47
Indirect Path c’: CTQ Total to PAI-BOR	0.02	-0.03 to 0.07	0.391	0.02

Note. CTQ = Childhood Trauma Questionnaire; BART-ET = Balloon Analogue Risk Task for Epistemic Trust; BSI-GSI = Brief Symptom Inventory – Global Severity Index. PAI-BOR = Personality Assessment Inventory – Borderline Features Scale. p < .05 = *; *p* < .01 = **; *p* < .001 = ***

**Table 4b:** Standardised estimates and 95% confidence intervals (CI) of each path in the SEM testing ETMCQ mistrust as a mediator between childhood trauma and borderline personality features, controlling for general psychological distress

Path	B	95% CI	*p*-Value	β
Path a: CTQ Total to ETMCQ Mistrust	0.07	0.01 to 0.13	0.022	0.18
Path d1: BSI-GSI to ETMCQ Mistrust	1.56	0.35 to 2.66	0.008	0.23
Path b: ETMCQ Mistrust to PAI-BOR	0.42	0.11 to 0.75	0.009	0.17
Path c: CTQ Total to PAI-BOR	0.21	0.06 to 0.38	0.009	0.21
Path d2: BSI-GSI to PAI-BOR	8.45	6.21 to 10.84	< 0.001	0.50
Indirect Path c’: CTQ Total to PAI-BOR	0.03	0.001 to 0.07	0.100	0.03

Note. CTQ = Childhood Trauma Questionnaire; ETMCQ = Epistemic Trust, Mistrust, and Credulity Questionnaire; BSI-GSI = Brief Symptom Inventory – Global Severity Index. PAI-BOR = Personality Assessment Inventory – Borderline Features Scale. p < .05 = *; *p* < .01 = **; *p* < .001 = ***

**Table 4c:** Standardised estimates and 95% confidence intervals (CI) for each path in the SEM testing BART-ET as a mediator between childhood trauma and both borderline personality features and general psychological distress

Path	B	95% CI	*p*-Value	β
Path a: CTQ Total to BART-ET	0.18	0.02 to 0.31	0.017	0.19
Path b1: BART-ET to PAI-BOR	0.42	0.27 to 0.59	<.001	0.38
Path b2: BART-ET to BSI-GSI	0.01	0.01 to 0.02	0.001	0.22
Path c1: CTQ Total to PAI-BOR	0.41	0.27 to 0.58	<.001	0.40
Path c2: CTQ Total to BSI-GSI	0.02	0.016 to 0.034	<.001	0.40
Indirect Path c1’: CTQ Total to PAI-BOR	0.08	0.009 to 0.143	0.028	0.07
Indirect Path c2’: CTQ Total to BSI-GSI	0.003	0.000 to 0.006	0.078	0.04

Note. CTQ = Childhood Trauma Questionnaire; BART-ET = Balloon Analogue Risk Task for Epistemic Trust; BSI-GSI = Brief Symptom Inventory – Global Severity Index. PAI-BOR = Personality Assessment Inventory – Borderline Features Scale. *p*< .05 = *; *p* < .01 = **; *p>*< .001 = ***

**Table 4d:** Standardised estimates and 95% confidence intervals (CI) for each path in the SEM testing ETMCQ Mistrust as a mediator between childhood trauma and both borderline personality features and general psychological distress

Path	B	95% CI	*p*-Value	β
Path a: CTQ Total to ETMCQ Mistrust	0.11	0.06 to 0.16	<.001	0.28
Path b1: ETMCQ Mistrust to PAI-BOR	0.68	0.34 to 1.04	<.001	0.27
Path b2: ETMCQ Mistrust to BSI-GSI	0.03	0.01 to 0.05	0.007	0.20
Path c1: CTQ Total to PAI-BOR	0.41	0.27 to 0.57	<.001	0.40
Path c2: CTQ Total to BSI-GSI	0.02	0.02 to 0.03	0.015	0.39
Indirect Path c1’: CTQ Total to PAI-BOR	0.08	0.03 to 0.14	0.005	0.08
Indirect Path c2’: CTQ Total to BSI-GSI	0.003	0.001 to 0.01	0.033	0.06

Note. CTQ = Childhood Trauma Questionnaire; ETMCQ = Epistemic Trust, Mistrust, and Credulity Questionnaire; BSI-GSI = Brief Symptom Inventory – Global Severity Index. PAI-BOR = Personality Assessment Inventory – Borderline Features Scale. *p* < .05 = *; *p* < .01 = **; *p* < .001 = ***

**Fig. 4a:**
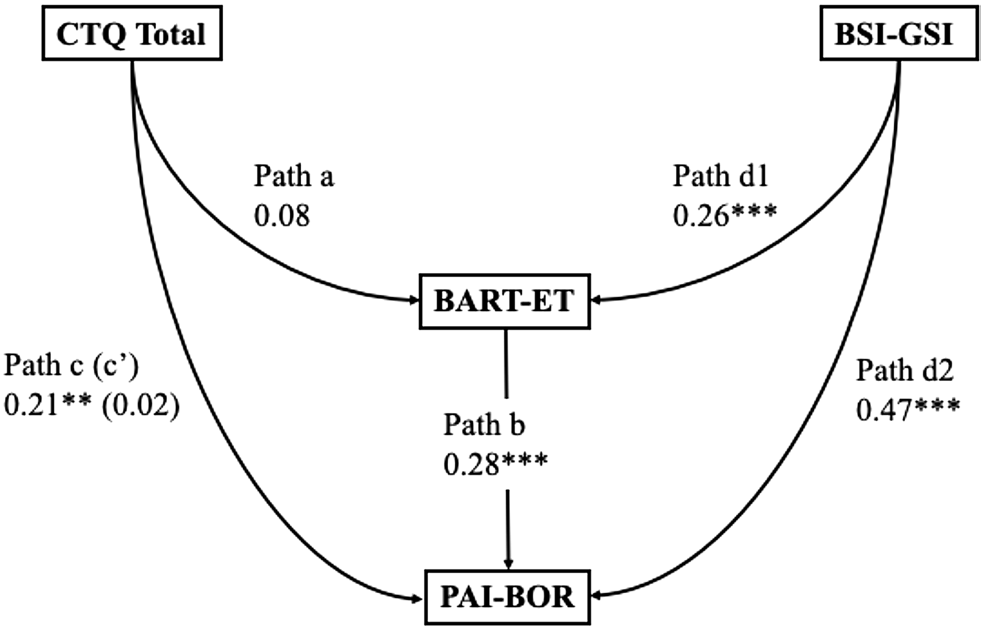
SEM testing BART–ET as a mediator between childhood trauma and borderline personality features, controlling for general psychological distress

**Fig. 4b:**
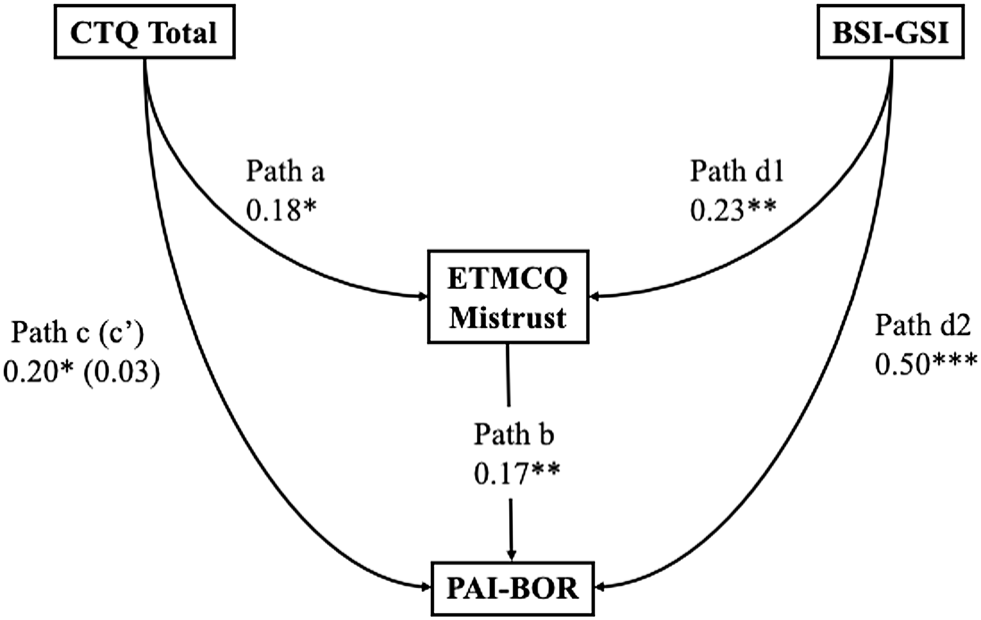
SEM testing ETMCQ Mistrust as a mediator between childhood trauma and borderline personality features, controlling for general psychological distress

**Fig. 4c:**
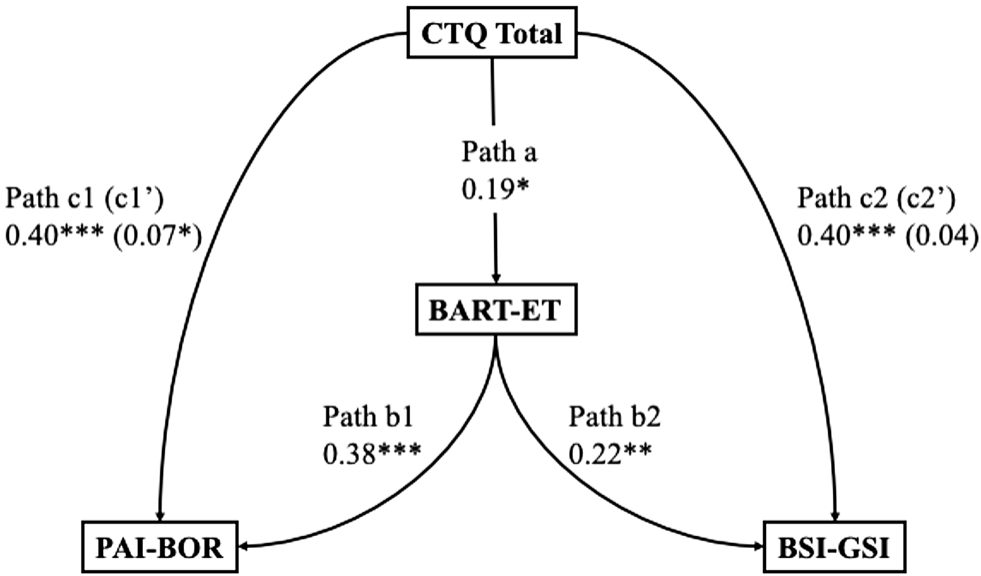
SEM testing BART-ET as a mediator between childhood trauma and both borderline personality features and general psychological distress

**Fig. 4d:**
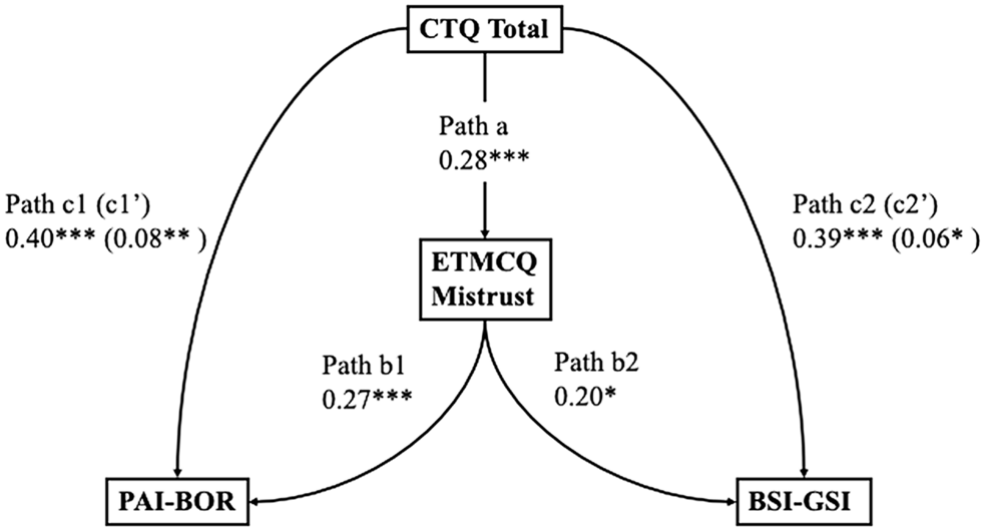
SEM testing ETMCQ Mistrust as a mediator between childhood trauma and both borderline personality features and general psychological distress

As shown in Table 4a and Fig. 4a, the preregistered mediation model with BART–ET as mediator tested whether behaviourally assessed epistemic mistrust explained the link between childhood trauma and BPD features while controlling for general distress. Childhood trauma did not significantly predict BART–ET (path a: β = 0.08, 95% CI [–0.09, 0.25], *p* = .370). The path from BART–ET to BPD features (path b) was significant (β = 0.28, 95% CI [0.18, 0.46], *p* < .001), indicating that participants who showed more behavioural mistrust tended to report higher BPD trait scores. However, the indirect effect of trauma on BPD via BART–ET was small and nonsignificant (β = 0.02, 95% CI [–0.03, 0.07], *p* = .391). The direct effect of trauma on BPD (path c′) remained significant (β = 0.21, 95% CI [0.08, 0.40], *p* = .008). General distress (BSI-GSI) significantly predicted both BART-ET (β = 0.26, *p* < .001) and BPD features (β = 0.47, *p* < .001), consistent with earlier correlations.

As shown in Table 4b and Fig. 4b, the preregistered mediation model with ETMCQ Mistrust as mediator indicated that childhood trauma significantly predicted self-reported mistrust (path a: β = 0.18, 95% CI [0.01, 0.13], *p* = .022), and mistrust significantly predicted BPD features (path b: β = 0.17, 95% CI [0.11, 0.75], *p* = .010). The indirect effect was small and non-significant (β = 0.03, 95% CI [–0.004, 0.06], *p* = .082). The direct effect of trauma on BPD remained significant (β = 0.21, *p* = .010). General distress again significantly predicted both mistrust (β = 0.23, *p* = .009) and BPD features (β = 0.50, *p* < .001).

Given these marginal findings, exploratory sensitivity analyses re-specified BSI-GSI as a parallel outcome rather than a covariate, allowing further examination of specificity to BPD traits. Two bivariate outcome SEMs were estimated: one with BART–ET as mediator and one with ETMCQ Mistrust as mediator. Both models were just-identified, so fit indices were not available.

As shown in Table 4c and Fig. 4c, when BART–ET was the mediator, childhood trauma significantly predicted BART–ET (β = 0.19, 95% CI [0.02, 0.31], *p* = .018). In turn, BART–ET predicted both BPD features (β = 0.38, 95% CI [0.26, 0.59], *p* < .001) and general distress (β = 0.22, 95% CI [0.09, 0.34], *p* = .001). The indirect effect of trauma on BPD via BART–ET was significant (β = 0.07, 95% CI [0.009, 0.14], *p* = .028), whereas the indirect effect on distress was nonsignificant (β = 0.04, 95% CI [–0.002, 0.09], *p* = .063). Direct effects of trauma on both BPD and distress remained significant (βs = 0.40, *p* < .001).

As shown in Table 4d and Fig. 4d, when ETMCQ Mistrust was the mediator, trauma significantly predicted mistrust (β = 0.28, 95% CI [0.06, 0.16], *p* < .001). Mistrust, in turn, predicted both BPD features (β = 0.27, 95% CI [0.34, 1.04], *p* < .001) and distress (β = 0.20, 95% CI [0.02, 0.05], *p* = .008). Indirect effects were significant for both outcomes: BPD (β = 0.08, 95% CI [0.03, 0.14], *p* = .005) and distress (β = 0.06, 95% CI [0.001, 0.01], *p* = .033). Direct effects of trauma on BPD and distress also remained significant (βs = 0.40 and 0.39, *p* < .001), indicating partial mediation.

Monte Carlo simulations (2,000 replications, *N* = 153) using the coefficients from Tables 4a–4d indicated that preregistered models had very low power to detect the observed small indirect effects (BART–ET: 9%; Mistrust: 13%). When accounting for measurement reliability (BART–ET α ≈ 0.80; ETMCQ Mistrust α = 0.55), simulated power estimates were lower in these models (BART–ET: 6%; Mistrust: 3%). In contrast, exploratory models had higher power to detect the larger indirect effects observed (BART–ET: 55%; Mistrust: 72%). These results suggest that null findings in preregistered models likely reflect low power and limited reliability, whereas exploratory models were sufficiently powered to detect larger mediation effects.

### Age, sex, and incentive as covariates

Age and sex were examined as potential covariates across both studies in exploratory analyses. Age was not significantly associated with any primary variables of interest, including epistemic stance measures (BART-ET, ETMCQ subscales), borderline personality features (PAI-BOR), childhood trauma (CTQ), or psychological distress (BSI-GSI). Accordingly, age was not included in any further analyses.

Sex showed a significant association with self-reported epistemic trust (*r* = –.32, *p* < .01), with males reporting lower trust levels than females. However, as epistemic trust was not significantly associated with other primary outcomes (e.g., PAI-BOR, CTQ, or BART-ET), sex was not retained as a covariate in subsequent models. These findings indicate that demographic variables did not meaningfully confound the key associations under investigation and were therefore excluded from the final analytic models.

Finally, even after accounting for differences in study compensation, participants who were more mistrustful (both behaviourally and by self-report) tended to show higher borderline personality features. Partial Spearman correlations adjusted for incentive showed that both behavioural mistrust (BART-ET) and self-reported mistrust (ETMCQ) were positively associated with BPD features (PAI-BOR): BART-ET–PAI-BOR, rₛ(partial) = 0.363, 95% CI [0.256, 0.462], *p* < .001; Mistrust–PAI-BOR, rₛ(partial) = 0.413, 95% CI [0.309, 0.507], *p* < .001; *n* = 273. These results indicate moderate associations that are robust to compensation differences.

## Discussion

This study examined how epistemic mistrust—operationalised through both a novel behavioural paradigm (the BART-ET) and established self-report measures (ETMCQ)—relates to borderline personality features, childhood trauma, and general psychological distress in young adults. Across two independent samples, greater epistemic mistrust was associated with higher borderline features and convergent links appeared between behavioural and self-reported mistrust. As expected, childhood trauma was associated with both mistrust and borderline traits, and exploratory mediation models suggested that mistrust measured both behaviourally and through self-report may partly account for this association. Importantly, however, these mediation results were provisional and must be interpreted cautiously given the cross-sectional design and low statistical power. Despite these limitations, taken together, provide further evidence for the role of epistemic mistrust—rather than simply reduced trust—as a distinctive cognitive-interpersonal process linking relational adversity with borderline vulnerability, while also demonstrating the potential of behavioural paradigms to capture these processes in vivo.

A key contribution of this study is the introduction of the BART-ET as a behavioural measure of epistemic mistrust [58]. The task adapts the Balloon Analogue Risk Task [30], traditionally used to assess risk-taking under uncertainty [31], to focus on how participants use or resist social information. Unlike classic BART measures of impulsivity and reward sensitivity [30, 31], the BART-ET reframes the task to quantify resistance to advice as an index of epistemic mistrust. This situates the concept of epistemic mistrust within the established decision-making literature while addressing a gap: most previous paradigms captured economic or affective decision biases, whereas the BART-ET task explicitly operationalised social information use, shifting the focus from monetary exchange or generic risk to advice-taking under uncertainty. In this sense, the present work extends prior experimental research on BPD and decision-making under uncertainty [8, 32], complementing findings that BPD traits are marked not only by risk-related decision-making biases [32] but also by enduring epistemic vigilance in contexts that typically promote cooperation and trust.

Regarding construct validity, the BART-ET showed expected associations with ETMCQ mistrust and with BPD features, while showing no relationship with self-reported trust—patterns consistent with convergent and discriminant validity. At the same time, these findings should be regarded as preliminary, given the absence of convergent behavioural tasks. The task may nonetheless capture variance not shared with questionnaires, plausibly reflecting real-time resistance to social influence and sensitivity to uncertainty—facets that self-reports often miss, consistent with well-known self-report/behaviour gaps [46, 47]. Taken together, these results suggest that the BART-ET holds promise as a complementary tool alongside self-report, but further validation is required to establish its psychometric robustness.

An important contribution of the current work is the demonstration that maladaptive epistemic stances—particularly mistrust, and to a lesser extent credulity—were more strongly associated with borderline personality features than the mere absence of trust. This asymmetry aligns with previous cross-sectional studies adopting the ETMCQ across community and clinical samples [6, 23, 49–51] and experimental and clinical observations that BPD is characterised by hypervigilance to potential threat, biased appraisal of social signals, and difficulties integrating positive interpersonal feedback [13]; even when symptoms remit, difficulties decoding positive cues persist [14]; and clinical syntheses emphasise heightened mistrust and expectations of harm rather than a unitary “deficit in affiliative trust” [43, 44]. This study reproduced this pattern in an advice context: participants higher on BPD features resisted largely accurate, benevolently framed guidance, suggesting a generalised and relatively inflexible stance. This is consistent with developmental accounts in which mistrust begins as an adaptation to adverse relational environments and later becomes entrenched and maladaptive when carried forward indiscriminately into safer contexts [2, 3].

Across both samples, borderline personality features were also associated with elevated epistemic credulity. Although this may seem paradoxical alongside heightened mistrust, developmental accounts anticipate that early adversity fosters oscillations between defensive scepticism and uncritical acceptance when epistemic calibration is unstable [6, 17]. Such fluctuations are consistent with theories of *hypermentalizing* in BPD, where heightened sensitivity to relational cues may lead to exaggerated or unstable interpretations of others’ intentions [17]. Empirical findings from trust games similarly demonstrate this interpersonal volatility: individuals with BPD often oscillate between ruptures and attempts at repair [8], or exhibit restricted but erratic cooperation patterns [12].

From the perspective of epistemic trust theory, both mistrust and credulity reflect expressions of a problematic epistemic stance, often co-occurring in individuals with instability in epistemic calibration. In the present task, however, behavioural deviation on the BART-ET most plausibly reflects epistemic mistrust—that is, resistance to advice even when it is likely accurate or benevolently framed. While the index may theoretically encompass multiple strategies (e.g., rejecting both accurate and inaccurate advice), its pattern of associations in the current data supports interpreting it primarily as a behavioural indicator of epistemic mistrust. In this light, mistrust and credulity may be understood not as opposites but as complementary maladaptive adaptations to uncertain or threatening interpersonal environments. Although further empirical research is needed to substantiate these assumptions, within developmental frameworks, this instability can be understood as the outcome of disrupted attachment and caregiving experiences, where children fail to develop a well-calibrated system for evaluating interpersonal reliability. Inconsistent caregiving may prevent the formation of stable expectations that others are generally safe to trust. Instead, in our view, children may adopt dual strategies: defensive mistrust to guard against harm, and sudden, indiscriminate openness to maximise opportunities for survival and receiving care. Carried into adulthood, these strategies may manifest as swings between excessive vigilance and uncritical acceptance, sustained by mood states or relational fears. Over time, these fluctuations may cause and be reinforced by ongoing psychopathology, which perpetuates mistrust and impedes the consolidation of secure, flexible epistemic trust.

The findings for childhood trauma align with theories linking early adversity to enduring interpersonal vulnerabilities [2] and with meta-analytic and recent survey evidence demonstrating robust associations between maltreatment, mistrust, and borderline symptomatology [21, 24, 27]. In Study 2, trauma was significantly associated with both behaviourally assessed and self-reported epistemic mistrust and borderline features, with emotional neglect and emotional abuse typically emerging as the strongest correlates [27]. The slightly stronger association observed for the ETMCQ Mistrust subscale may partly reflect shared method variance, as both CTQ and ETMCQ rely on self-report data. This pattern converges with developmental accounts emphasising that disruptions in early caregiving environments sensitise individuals to potential interpersonal threat, leaving enduring imprints on social–cognitive processes. By contrast, trauma showed limited associations with self-reported epistemic trust and epistemic credulity in this study. Several explanations are plausible: reliance on retrospective trauma reporting and the non-clinical nature of the sample. Exploratory subscale contrasts provided tentative signals—for example, elevations linked specifically to histories of physical and emotional maltreatment—but these effects were conservative after correction, reinforcing that such patterns must be replicated in larger and more diverse samples.

Turning to mediation, preregistered models and exploratory sensitivity analyses suggest that behavioural and self-report measures mediated the trauma → BPD association, and that these associations are likely to be specific to BPD, but rather reflect vulnerability to distress in general, consistent with recent theoretical models [48]. Given the modest effect sizes, low statistical power for preregistered models, and reliability limitations of ETMCQ Mistrust subscale, these mediation findings must be regarded as provisional and hypothesis-generating, not causal. Nevertheless, the pattern is consistent with the theoretical proposal that epistemic mistrust may constitute a mechanism through which childhood adversity confers vulnerability to both borderline symptomatology and more general psychological distress.

An additional consideration is the relationship between distress and epistemic stance. Consistent with prior work [6], distress was strongly associated with both behavioural and self-reported mistrust, as well as with credulity. Yet the direction of this association is unlikely to be unidirectional. On one hand, heightened distress may amplify vigilance to social threat and bias interpretations of others’ intentions, thereby fuelling disruptions in the form of mistrust and credulity [13, 15, 43]. On the other hand, entrenched mistrust and credulity may contribute to distress over time—by impeding the receipt of social support, exacerbating interpersonal conflict, reinforcing expectations of unreliability, and increasing vulnerability to exploitation and manipulation [3, 6, 44, 45]. This bidirectional reciprocity is well recognised in broader psychopathology research, which links social–cognitive dysfunctions to emotional suffering across multiple diagnostic categories [25]. Moreover, clinical evidence suggests that reductions in mistrust track symptom improvement during psychotherapy, reinforcing the idea that mistrust and distress are dynamically linked [52–54]. Thus, epistemic disruptions and distress may form a mutually reinforcing cycle that sustains vulnerability over time, a possibility that future longitudinal and experimental work should directly test.

Finally, exploratory analyses revealed sex-linked differences in epistemic stance: men reported significantly lower levels of epistemic trust compared to females. This pattern is broadly consistent with prior ETMCQ validation work, which found men to show lower trust and higher mistrust and credulity relative to women, albeit with small effect sizes [6]. Importantly, in the present data, sex differences did not extend to epistemic mistrust, credulity, BART-ET, or borderline features. These findings should be interpreted cautiously given the small male subsample and the predominantly Asian, female composition of the two independent samples, which reflects the demographic profile of psychology majors at the host university. Cultural and gendered norms may further shape both self-report and advice-taking behaviour. More broadly, although BPD is more often diagnosed in women, evidence suggests possible under-recognition in men, raising the possibility that sex-linked variations in epistemic stance may contribute differently to the manifestation or detection of borderline traits across genders [55]. Together, these findings highlight the need for future research to examine sex, culture, and context more systematically in relation to epistemic stance.

### Limitations and future research

Several limitations should be considered when interpreting these findings. First, although participants were drawn from a non-clinical university population, a meaningful subset scored above clinical thresholds for borderline personality features and psychological distress. Future research should establish whether the same patterns hold in clinical populations, where epistemic difficulties may be more pervasive or entrenched. In addition, the sample was predominantly Asian and female psychology undergraduates, reflecting the demographic composition of the host programme. This composition limits generalisability, particularly in relation to sex, gender, cultural, and socio-economic influences on advice-taking and epistemic stance. Replication across more diverse populations—including different ethnic, cultural, and educational groups, as well as across different developmental stages—will be crucial for testing the robustness and generalisability of these findings.

Second, measurement limitations warrant caution. The ETMCQ Mistrust subscale showed internal consistency values ranging from α = 0.55 to 0.66 in the present samples. Reported estimates in the broader literature also vary, ranging from 0.51 to 0.73 across community samples (e.g [24]: ω = 0.51; [50]: α = 0.57; [6]: α = 0.65–0.72; [49]: α = 0.67; [56]: α = 0.67; [23]: ω = 0.73), indicating that measurement error may be a common issue when operationalising epistemic mistrust, particularly in non-clinical samples, and highlights the importance of ongoing refinement and validation of this subscale [57]. For the behavioural task, the BART-ET is a novel paradigm whose construct validity remains preliminary. Although the present findings offer initial support for convergent and discriminant validity, stronger validation requires the inclusion of convergent behavioural tasks, test–retest designs, and trial-level modelling. Notably, the current scoring method calculates only the absolute discrepancy from the advised pump number, collapsing overshooting (active opposition) and undershooting (cautious withdrawal). These may reflect distinct mistrust strategies that future work should disaggregate through refined metrics such as trial-level mixed models or calibration indices. Future work will incorporate direction-specific deviations, advice-accuracy calibration, and computational advice-weight models to more precisely differentiate mistrust from credulity.

Third, the BART-ET in its current form cannot reliably distinguish epistemic trust from credulity. Participants who closely followed advice may have done so out of openness (adaptive trust) or indiscriminate acceptance (maladaptive credulity). Yet, the version of the BART-ET used in this study was not designed to capture such differences as without varying advice reliability, these distinctions cannot be established. Future versions should introduce conditions with systematically misleading or unreliable advice to test sensitivity to context and distinguish adaptive from maladaptive openness.

Fourth, the design did not include manipulation checks (e.g., perceived credibility of advice, emotional responses to advice, or subjective affect), which may limit interpretability of observed discrepancies. Additionally, while Study 2 included financial incentives and Study 1 did not, these differences were modelled and did not substantively alter the findings. Even so, future work should standardise incentive structures to remove potential confounds.

Fifth, the cross-sectional design precludes causal inference. Although exploratory mediation analyses were consistent with the hypothesis that epistemic mistrust may partly account for the link between childhood trauma and borderline features, these findings remain tentative. Longitudinal and experimental studies will be required to clarify whether epistemic mistrust functions as a stable mediator, a context-sensitive adaptation, or both. Power limitations should also be acknowledged: Monte Carlo simulations indicated that the study was underpowered to detect small indirect effects, and preregistered SEMs were just-identified, further constraining the strength of mediation conclusions.

Finally, this study did not incorporate full design-level extensions that would strengthen the validity of the BART-ET, such as computational advice-weight modelling, trial-level mixed-effects analyses, convergent validity testing with other behavioural paradigms, or test–retest reliability assessments. These limitations were deliberate, as our goal here was to introduce and apply the BART-ET within a broader test of epistemic stance, whereas methodological refinements and psychometric validation will be the focus of a separate, dedicated report.

## Conclusions

In sum, this study highlights epistemic mistrust as a central process linking early relational adversity with borderline personality vulnerability. Across two independent samples, mistrust—more than the mere absence of trust—was robustly associated with borderline features and complemented by evidence of elevated credulity, underscoring the need to consider both processes within developmental models of personality vulnerability. By introducing the BART-ET, we extend epistemic trust research into the decision-making domain, offering a novel behavioural index of real-time resistance to advice under uncertainty. The BART-ET showed unique predictive value beyond self-report, reinforcing the benefit of multi-method approaches that capture epistemic stance both through reflective questionnaires and in-the-moment interpersonal behaviour. At the same time, limitations in reliability, construct validity, and generalisability mean that these findings must be interpreted with caution. We therefore regard this work as an initial step: it demonstrates the promise of behavioural paradigms such as the BART-ET and further highlights the importance of distinguishing mistrust from both trust and credulity. It points toward the value of longitudinal, experimental, and design-level extensions—including manipulations of advice reliability and computational modelling—to clarify the causal role of epistemic stance in psychopathology. Taken together, our results support the growing view that epistemic mistrust represents not merely a deficit in affiliative openness, but a maladaptive interpersonal adaptation that may both stem from and perpetuate psychological vulnerability.

## Data Availability

The data from this study are not publicly available, as consent for data sharing was not included in the participant consent form. However, the laboratory task (BART-ET) can be made available upon request by contacting the authors, and we have uploaded the R scripts and session information to OSF to ensure transparency (https://osf.io/dhm89/files/osfstorage).

## Declarations

### General scientific summary

This research introduces a new behavioural task—the BART-ET—to measure how open people are to trusting advice from others. Across two studies with young adults, we found that individuals with more borderline personality traits were less likely to follow interpersonal advice, especially if they had experienced childhood trauma. This reluctance was linked to a general mistrust of others’ knowledge rather than low trust alone. Our findings suggest that epistemic mistrust may play an important role in personality difficulties and that behavioural tasks like the BART-ET can offer new ways to study social thinking in clinical contexts.

## Abbreviations

BART: Balloon Analogue Risk Task
BART-ET: Balloon Analogue Risk Task – Epistemic Trust version
BPD: Borderline Personality Disorder
PAI-BOR: Personality Assessment Inventory – Borderline Features Scale
ETMCQ: Epistemic Trust, Mistrust, and Credulity Questionnaire
CTQ: Childhood Trauma Questionnaire
BSI: Brief Symptom Inventory
BSI-GSI: Brief Symptom Inventory – Global Severity Index
SEM: Structural Equation Modelling
SD: Standard Deviation
M: Mean
N: Sample size (number of participants)
